# The Role of Insulin Receptor Isoforms in Diabetes and Its Metabolic and Vascular Complications

**DOI:** 10.1155/2017/1403206

**Published:** 2017-10-19

**Authors:** O. Escribano, N. Beneit, C. Rubio-Longás, A. R. López-Pastor, A. Gómez-Hernández

**Affiliations:** ^1^Biochemistry and Molecular Biology II Department, School of Pharmacy, Complutense University of Madrid, Madrid, Spain; ^2^Health Research Institute of San Carlos Clinic Hospital (IdISSC), Madrid, Spain; ^3^CIBER of Diabetes and Associated Metabolic Diseases, Madrid, Spain

## Abstract

The insulin receptor (IR) presents by alternative splicing two isoforms: IRA and IRB. The differential physiological and pathological role of both isoforms is not completely known, and it is determinant the different binding affinity for insulin-like growth factor. IRB is more abundant in adult tissues and it exerts mainly the metabolic actions of insulin, whereas IRA is mainly expressed in fetal and prenatal period and exerts mitogenic actions. However, the change in the expression profile of both IR isoforms and its dysregulation are associated with the development of different pathologies, such as cancer, insulin resistance, diabetes, obesity, and atherosclerosis. In some of them, there is a significant increase of IRA/IRB ratio conferring a proliferative and migratory advantage to different cell types and favouring IGF-II actions with a sustained detriment in the metabolic effects of insulin. This review discussed specifically the role of IR isoforms as well as IGF-IR in diabetes and its associated complications as obesity and atherosclerosis. Future research with new IR modulators might be considered as possible targets to improve the treatment of diabetes and its associated complications.

## 1. Introduction

Type 2 diabetes is one of the greatest global health emergencies of the twenty-first century. It is currently estimated that there are 415 million people with diabetes in the world and another 318 million with impaired glucose tolerance, which puts them at high risk of developing the disease. It is expected that by 2040 there will be 642 million people living with this disease [1].

Type 2 diabetes is characterized by an increase in glycaemia and is associated with several complications, mainly cardiovascular, that reduce quality and life expectancy. In type 2 diabetes, insulin resistance is considered the initial event leading to the development of the disease. Resistance to insulin action results in decreased glucose uptake by peripheral tissues, as well as in lipid homeostasis alterations. Several tissues play a key role in the development of insulin resistance and type 2 diabetes.

In general, the regulation of insulin-mediated glucose metabolism in peripheral tissues via IRS/PI3K/Akt signalling pathway is key in this disease. Proteins involved in this signalling pathway play an important role in the molecular mechanisms that lead to the development of insulin resistance. Thus, individuals with insulin resistance in which the expression of the IR was reduced or absent were identified [2, 3]. Insulin resistance-associated insulin receptor substrate-1 (IRS-1) mutations have also been described [4], as well as increased phosphorylation of this substrate on serine residues [5], which leads to a decrease in its tyrosine phosphorylation and, consequently, to less insulin signalling. In the insulin signalling pathway, the first critical node is the receptor itself (IR), which presents two isoforms (IRA and IRB) by alternative splicing. In recent years, it has been described that the expression profile of both isoforms could be altered in pathological situations, including diabetes. This review discussed specifically the role of IR isoforms as well as IGF-IR in diabetes and its complications as obesity and atherosclerosis.

## 2. Insulin and Insulin-Like Growth Factors

Insulin is a 51 amino acid polypeptidic hormone, discovered in 1922 by Banting et al. [6]. It is synthesized by *β*-cells in pancreatic islets as a single chain of 110 amino acids called preproinsulin. Through proteolytic enzymes, preproinsulin loses the signal peptide of the amino-terminal end giving rise to proinsulin. Subsequently, cleavage of an inner fragment of proinsulin (C-peptide) gives rise to insulin with two chains (*α* and *β*), which are joined by two disulfide bridges, and a third intracatenary bridge in the *α* chain [7].

Insulin-like growth factors (IGF-I and IGF-II) are single chain polypeptides with structural homology to proinsulin. They regulate proliferation and differentiation processes in a multitude of cell types and, in addition, are able to exert metabolic effects similar to those of insulin [8]. Although circulating levels of IGF-I and IGF-II are mainly determined by their hepatic production, most of the cells synthesize these growth factors. The bioavailability of IGFs depends on their binding to IGFBPs, of which six different ones have been identified in humans [9]. IGFBP-3 is responsible for transporting more than 90% of circulating IGFs, forming a ternary complex with an additional protein called the acid-labile subunit that limits the extravascular transit of IGFs. At the cellular level, IGFBPs form a binary complex with IGFs that crucially modulate their local actions [10].

## 3. Insulin Receptor Isoforms

**Figure 1:**
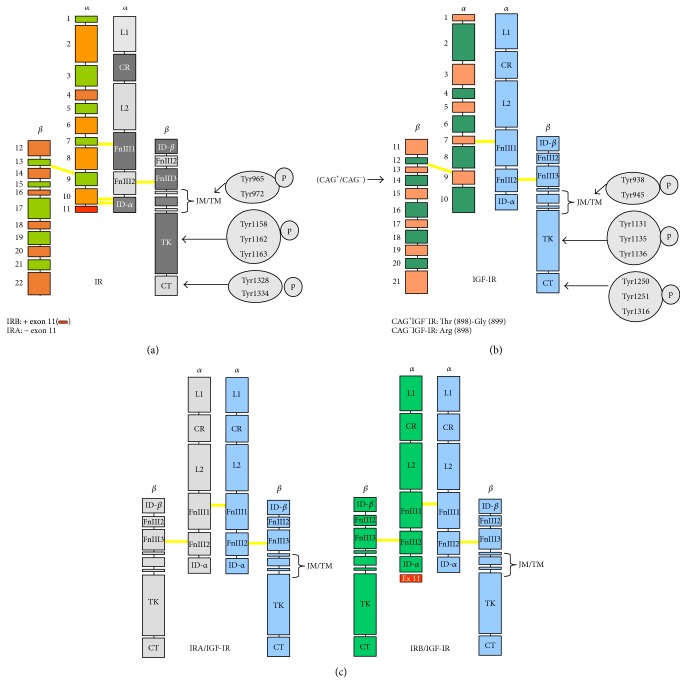
Structure and phosphorylation residues of IR, IGF-IR, and hybrid receptors. (a) Schematic representation of IR. (Left) *α* chain: exons 1–10 (IRA) or exons: 1–11 (IRB) and *β* chain: exons 12–22. (Right) Domains of IR: L1, large domain rich in Leu; CR, domain rich in Cys; L2, large domain 2; Fn, fibronectin III type domain; TM, transmembrane domain; JM, juxtamembrane domain; TK, Tyr kinase domain; CT, C-terminal domain. In the JM, TM, TK, and CT domains, Tyr phosphorylation residues are indicated. (b and c) Schematic representations of IGF-IR isoforms (CAG^+^/CAG^−^) and hybrid receptors (IRA : IGF-IR and IRB : IGF-IR). In the figure are also indicated the different domains and Tyr phosphorylation residues.

The IR is a heterotetramer formed by two extracellular *α* subunits and two transmembrane *β* subunits linked by disulfide bridges [11]. The *α* chain and 194 residues of the *β* chain form the extracellular portion, and a single chain of 403 residues of the *β* chain constitutes the cytoplasmic domain responsible for the receptor's tyrosine kinase activity (Figure 1(a)). Similar to insulin, the two IR chains are derived from the same precursor, the proreceptor that is assembled after a proteolytic breakdown.

A 22-exon gene that is located on chromosome 19 encodes the human IR. The alternative splicing of exon 11 in its primary transcript gives rise to the two IR isoforms [12]. These two isoforms differ in a sequence of 12 amino acids at the C-terminus of the *α*-chain, which is absent in IRA and present IRB (Figure 1(a)). Although IR has a common structure in all vertebrates, with up to 20% invariability, alternative splicing of exon 11 is an exclusive feature of mammals [13].

**Figure 2:**
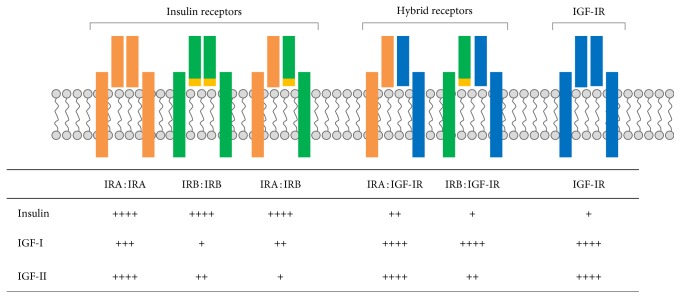
Insulin and IGFs receptor family. Each receptor binds insulin, IGF-I, or IGF-II with a different affinity. Ligand affinities are showed as + (very low affinity), ++ (low affinity), +++ (high affinity), and ++++ (very high affinity).

The relative expression of the two isoforms is tissue-specific [14]. In adult life, IRB is expressed predominantly in insulin target tissues, such as liver, muscle, adipose tissue, and kidney. In contrast, the expression of IRA is predominant in fetal and tumor tissues [15], although it can also be found in most adult tissues. In the literature, we find discrepancies on the affinity of IR isoforms for insulin. Different studies indicate a greater affinity of insulin for IRA than IRB, Kd = 0.91 ± 0.3 nM for IRA expressing CHO cells versus Kd = 1.56 ± 0.5 nM for IRB expressing CHO cells [16]. In the same way, Yamaguchi et al. found a 2-fold higher affinity for insulin in Rat1 cells expressing IRA than those expressing IRB [17]. However, Whittaker et al. found identical affinities for both isoforms in 293T cells, Kd = 2.1 ± 0.2 nM for IRA-expressing cells versus Kd = 1.8 ± 0.2 nM for IRB-expressing cells [18] (Figure 2()). The differences found in the affinity for insulin are, in any case, very modest. Much clearer is the greater affinity of IRA for IGF-II (Figure 2()), being much larger than for IGF-I [19]. The C and D domains of IGF-II appear to be important in these affinity differences [20].

## 4. Signal Transduction

Insulin binding to the *α* subunit of IR induces a conformational change that facilitates the binding of ATP, phosphorylation of *β* subunits, recruitment of intracellular substrates, and their subsequent phosphorylation. In the insulin-free state, the IR inhibitory conformation maintains a separation between the two tyrosine kinase domains that prevents the activation loop of one of them by reaching the catalytic site of the other [21]. A single insulin molecule binds the two extracellular domains by reducing the gap between the tyrosine kinase domains, allowing the activation loops of the tyrosine kinase domains.

The receptor tyrosine kinase activity phosphorylates numerous intracellular substrates [22], the best characterized are the IRSs. The IRSs family is formed by six members with high homology and different tissue distribution and function. IRS-1 and IRS-2 are widely distributed, with IRS-2 being fundamental in *β*-cells and hepatocytes, and IRS-1 in endothelial and vascular smooth muscle cells (VSMCs). When Tyr-phosphorylated, IRSs serve as anchor points for proteins with SH2 (Src homology 2 domain) domains. Many of these proteins act as adapter molecules, such as the p85 regulatory subunit of PI3K or Grb2 (growth factor receptor-bound protein 2). In addition to IRSs, IR phosphorylates in tyrosine residues several other substrates [23]. For example, Shc proteins are phosphorylated by IR resulting in the activation of the Ras/MAPK pathway. Grb2-associated binder (GAB) proteins are also substrates for a variety of receptors, including IR. Cbl is also an IR substrate that recruits other proteins, such as the Cbl-associated protein (CAP) to the insulin-signalling pathway participating in the control of insulin-stimulated glucose uptake [24]. Finally, Crk family adaptors are widely expressed and mediate formation of signalling complexes via their SH2 and SH3 domains in response to a variety of extracellular stimuli [25]. Crk-L and CrkII were also reported to interact with and be phosphorylated by both IR and IGF-IR [26–28]. In this complex cascade of signals, the two main traditionally recognized pathways mediate metabolic and mitogenic effects by activation of PI3K or Ras, respectively.

### 4.1. PI3K Pathway

PI3K functions as a critical node in insulin signalling. It is formed by a regulatory subunit and a catalytic subunit, which have different isoforms. The interaction of the SH2 domain of the p85 regulatory subunit with tyrosine-phosphorylated IRS domains results in the activation of the p110 catalytic subunit [29]. Once activated, PI3K catalyzes the phosphorylation of membrane phospholipid PIP_2_ (phosphatidylinositol-4,5-bisphosphate) to form PIP_3_ (phosphatidylinositol-3,4,5-triphosphate). PIP_3_ acts as a second messenger and allows the anchoring in the plasma membrane and activation of proteins with PH (pleckstrin homology) domains, like PDK1 (phosphoinositide-dependent protein kinase 1). Activation of PDK1 leads to phosphorylation of Akt in threonine 308 and of PKC*ζ* in threonine 410. For complete activation of Akt, phosphorylation of serine 473 is also necessary, which is not mediated by PDK1, but by mTORC2 [30]. Although the most important role of mTORC2 is likely the phosphorylation of Akt, [31], mTORC2 as occurs with PDK1, is able to phosphorylate some PKC family members such as PKC*α*, a regulator of the actin cytoskeleton [31, 32]. Moreover, mTORC2 is also able to phosphorylate PKC*δ* [33] and PKC*ζ* [34], as well as PKC*γ* and PKC*ϵ* [35], all of which regulate several aspects of cytoskeletal remodeling and cell migration.

Activation of Akt by PDK1 and mTORC2 allows the phosphorylation and activation of many downstream targets, such as glycogen synthase kinase 3 (GSK3) and phosphofructokinase-2 (PFK2), and is involved in glucose metabolism by inducing GLUT4 translocation from intracellular compartments to the plasma membrane [36]. Activated Akt also phosphorylates FoxO proteins (FoxO1, FoxO3, and FoxO4), inhibiting their action by promoting their nuclear exclusion and degradation. FoxO proteins act as transcription factors regulating several cellular functions, including metabolic actions, stimulation of apoptosis, and inhibition of cell growth [37].

Another pathway regulated by the activation of PI3K-Akt is raptor-mTOR pathway (mTORC1), which regulates cell metabolism and growth and integrates signals from insulin and growth factors with those derived from nutrients [22]. Akt phosphorylates and inactivates TSC2 (tuberous sclerosis complex 2), which is forming a heterodimeric complex with TSC1 (tuberous sclerosis complex 1) functioning as a GTPase Rheb (Ras-homolog enriched in brain) activator [36]. Inactivation of TSC2 by Akt reduces the GAP activity (GTPase activating protein) of the TSC1/TSC2 complex, thereby increasing the amount of GTP bound to Rheb, which causes the activation of mTORC1. This complex is the main regulator of protein synthesis and ribosomal biogenesis through phosphorylation of 4E-BP1 (eukaryotic translation initiation factor 4E-binding protein 1) and p70S6 kinase (p70S6K). 4E-BP1 binds to the translation initiator factor eIF4E, repressing protein synthesis. Activation of mTORC1 induces the phosphorylation of 4E-BP1 in multiple residues, releasing eIF4E, which binds to the translation initiation complex and allows protein synthesis [38]. p70S6K is a serine/threonine kinase that directly phosphorylates the S6 ribosomal protein and activates, by phosphorylation or interaction, multiple proteins from the mRNA translation machinery, collectively affecting both initiation and elongation of protein translation [39].

### 4.2. Ras-MAPK Pathway

The Ras-MAPK pathway is activated by insulin after the binding of Grb2 to phosphorylated IRSs or SH2 domain proteins (Shc and GAB1). Grb2, through one of its two SH3 domains, binds and activates the SOS guanine nucleotide exchanger factor. This causes activation of the Ras GTPase domain, and the subsequent activation of Raf, which triggers a signalling cascade resulting in the phosphorylation of MEK1/2 and ERK1/2 [40].

In its inactive form, ERK1/2 is mainly located in the cytoplasm where it is forming a heterodimer with MEK1/2. When ERK1/2 is phosphorylated, it dissociates from MEK1/2 and translocates to the nucleus, where it phosphorylates a large variety of substrates involved in the activation of a complex transcriptional program. In addition, active ERK1/2 also phosphorylates numerous substrates in the cytoplasmic compartment.

To enhance the complexity, it has been shown that the signalling pathways vary depending on the activated IR isoform. For instance, in pancreatic *β*-cell lines, glucokinase (GK) gene transcription is promoted by insulin through IRB/PI3K class II-like activity/Akt, whereas insulin expression is regulated through IRA/PI3K class Ia/p70S6K [41]. Moreover, in NIH3T3 fibroblasts, activation of IRA by pp120 undergoes faster internalization and recycling than IRB [42, 43] and is differentially regulated by IGF-II and insulin [44]. In addition, IGF-II binding to IRA is associated with increased cell proliferation and invasion and nuclear IRS-1 translocation [19, 45], whereas IRB, which does not bind to IGF-II, is associated with differentiation and metabolic signals following insulin stimulation [45].

In addition to the classical signalling pathways, many evidences have indicated a role in the nucleus for many receptor tyrosine kinases, including the IR and IGF-IR [46, 47]. These data showed nuclear import and a direct transcriptional role for both IR and IGF-IR adding a new layer of complexity. Even, it has been described that nuclear envelope is the major binding site for insulin [48]. Therefore, the ability of IR and IGF-IR to function as transcription factors, although poorly understood, constitutes a new paradigm in signal transduction [47]. Moreover, enhanced IR translocation to the nuclei was associated with increased expression of malic enzyme, suggesting a role of nuclear IR in the phosphorylation of insulin response element (IRE) transcription factors [49]. In the same way, it has been described that nuclear IR regulates the transcription of early growth response 1 (egr-1) and glucokinase (GK), which regulate mitogenic and metabolic responses, respectively [50].

## 5. IGF-I Receptor and Hybrid Receptors IR/IGF-IR

IGF-I receptor (IGF-IR) belongs, along with the IR, to the class II receptor tyrosine kinase superfamily. Both receptors have a high structural homology ranging from 45 to 65% in the ligand-binding domains and 65 to 85% in the tyrosine kinase and substrate recruitment domains [51, 52]. IGF-IR is widely expressed in most tissues and regulates important cellular processes such as differentiation, cell growth, and apoptosis [53]. Both IGF-I and IGF-II interact with IGF-IR, although IGF-I binds with much greater affinity than IGF-II (Figure 2). In human aortic smooth muscle cells, both IGF-I and IGF-II activate IGF-IR and/or IR/IGF-IR at physiological concentrations [54]. Ligand binding to the extracellular *α*-chain of IGF-IR leads to the autophosphorylation of three tyrosine residues in the tyrosine kinase domain of the *β*-chain (Figure 1(b)), which activates signalling pathways similar to those described for IR. Recently, it has been reported that changes in the affinities were also reflected in the IR phosphorylation pattern, meaning that position 718 is important for IGF affinity and activation of both IR isoforms, whereas mutations in position 718 did not affect insulin affinity [55].

IR and IGF-IR are on the plasma membrane as preformed dimers composed of two *αβ* subunits linked by disulfide bridges. Dimerization of the two subunits takes place in the endoplasmic reticulum, prior to the proteolytic breakdown of the proreceptor that originates the *α*- and *β*-chains [56]. A consequence of the high degree of homology between the two receptors is the formation of hybrid receptors composed of an *αβ* subunit of IR (IRA or IRB) and an *αβ* subunit of IGF-IR (Figure 1(c)).

Kasuga et al. first proposed the existence of IR/IGF-IR hybrid receptors in 1983 [57]. Six years later, Soos and Siddle identified hybrid receptors from human placenta [58], thus confirming their existence. It is now known that hybrid receptors are widely distributed in most tissues and cell types of mammals [59], including vascular cells such as endothelial cells [60] and VSMCs [61–63]. It is believed that heterodimerization of the two receptors occurs with similar efficiency to homodimerization, so the proportion of hybrid receptors depends on the relative abundance of individual receptors [59]. The three ligands (insulin, IGF-I, and IGF-II) are able to bind and activate, in addition to their own receptors, IRA/IGF-IR and IRB/IGF-IR hybrid receptors, albeit with different affinity and efficacy. It has been described that IRA/IGF-IR and IRB/IGF-IR hybrids bind insulin with similar relatively low affinity, which was intermediate between that of homodimeric IR and homodimeric IGF-IR. However, both IRA/IGF-IR and IRB/IGF-IR hybrids bound IGF-I and IGF-II with high affinity, at the level of homodimeric IGF-IR [64] (Figure 2).

## 6. IGF-II Receptor (IGF-IIR)

The IGF-II receptor (IGF-IIR), which is homologous to the cation-independent mannose-6-phosphate receptor, has a high affinity for IGF-II [65]. It is a type I transmembrane glycoprotein composed of a long extracellular region, a small transmembrane region of 23 amino acids, and a cytoplasmic tail of 167 amino acids.

IGF-IIR regulates the amount of circulating and tissue IGF-II by transporting it into the cell and degradation. Since IGF-II promotes cell growth, differentiation, and survival, primarily through IGF-IR and IRA, IGF-IIR acts as a growth inhibitor by decreasing the bioavailability of IGF-II [66]. However, it is a multifunctional receptor that, in addition to IGF-II, binds lysosomal enzymes labelled with mannose-6-phosphate, allowing the transfer of newly synthesized lysosomal enzymes from the trans-Golgi network to the late endosomes. It also binds other proteins containing mannose-6-phosphate moieties such as the latent form of TGF-*β*, granzyme B, urokinase-type plasminogen activator receptor (uPAR), plasminogen, glycosylated leukaemia inhibitory factor (LIF), or retinoic acid. Activation of TGF-*β* is inhibited by mannose-6-phosphate and it has been proposed that such activation requires the formation of a complex between IGF-IIR, plasminogen, uPAR, and TGF-*β*. This model suggests that urokinase, linked to uPAR, transforms plasminogen into plasmin, which activates the latent form of TGF-*β*. Active TGF-*β* stimulates cellular apoptosis through the activation of its receptors. IGF-IIR has been considered a tumor suppressor receptor [67, 68], as it regulates the intracellular uptake of IGF-II, lysosomal enzymes, glycosylated LIF, and granzyme B (involved in apoptosis induced by cytotoxic T lymphocytes) as well as the activation of TGF-*β*; all of these processes require a strict control to avoid carcinogenesis.

Although it has classically been considered an IGF-II clearance receptor, a few groups have reported that IGF-IIR is also able to trigger intracellular signalling in response to IGF-II regulating cell behaviour [69–74]. For example, IGF-IIR, activated by IGF-II, interacts with G*α*q protein and induces hypertrophy and apoptosis in cardiomyoblasts and myocardic cells [75, 76], suggesting a role of IGF-II and its receptor in cardiac cell turnover. More efforts are needed in order to further characterize the role of IGF-IIR as a signalling protein and the signalling cascades involved.

## 7. Role of Insulin Receptor Isoforms in Type 2 Diabetes and Related Metabolic Complications

Insulin receptor splicing is a conserved mechanism in mammals, responsible for the specificity in insulin signalling and IGFs. Thus, a predominant expression of IRA is associated with a decrease in the metabolic signalling of insulin and an increase in the signalling of IGFs, being of great importance in development and fetal growth. In contrast, increased expression of IRB is associated with a predominance of metabolic actions of insulin during adult life. Dysregulation of this mechanism with an increase of IRA in adult life may play an important role in different pathological processes [77]. In 1999, Frasca et al. [19], Sciacca et al. [78], and colleagues demonstrated for the first time the differential expression of IR isoforms in tumor cells compared to normal cells. A predominant expression of the IRA isoform has been described thereafter in a wide variety of cancers, including lung, colon [19], ovarian [79], thyroid [80], and muscle cancer [81].

### 7.1. Insulin Resistance and Glucose Metabolism

The role of IRA/IRB ratio alterations in the development of insulin resistance is already poorly understood. An increase in the IRA/IRB ratio in insulin resistance in different insulin target tissues has been shown [82–84]. However, other authors have found no significant alterations in IRA/IRB ratio in different models of insulin resistance [14]. In adipocytes and skeletal muscle from diabetic patients, some studies have reported a decrease in the IRA/IRB ratio [16, 85–87], whereas other authors did not find any difference [88–90]. Indeed, in spontaneously obese diabetic rhesus monkeys, Huang et al. found an increased IRA/IRB ratio in the liver and muscle of these animals suggesting that hyperinsulinemia could regulate the alternative splicing of IR mRNA favouring insulin resistance [83, 84]. This controversy may be because type 2 diabetes is a complex and heterogeneous syndrome, so that IR splicing may be affected by different variables such as hyperinsulinemia, hyperglycemia, age, stage of the disease, and genetic alterations.

**Figure 3:**
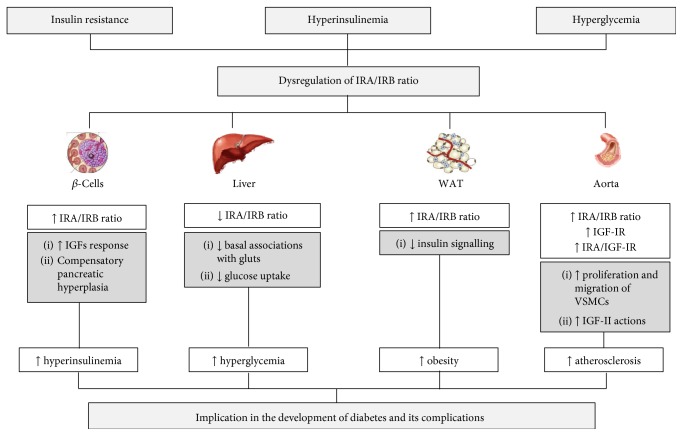
Dysregulation of IR isoforms in type 2 diabetes and its related complications. It has been described in insulin target tissues such as liver, white adipose tissue, or aorta that a significant increase of IRA/IRB ratio might be involved in a decreased glucose uptake, less insulin signalling, and an increased growth of atherosclerotic plaques, respectively. Moreover, in pancreatic beta cells might be implicated the compensatory hyperplasia that occurs in insulin-resistant states. These mechanisms contribute to hyperglycemia, hyperinsulinemia, obesity, and vascular complications.

In pancreatic-beta cells, it has been reported that the profile of IR isoform expression might be modified by chronic hyperglycemia and/or liver insulin resistance (Figure 3). In this regard, chronic hyperglycemia has been associated with increased IRA/IRB ratio in human pancreatic islets [91]. In mouse beta-cell lines, IRA, but not IRB, conferred a proliferative capacity in response to insulin or IGF-I, providing a potential explanation for the beta-cell hyperplasia induced by liver insulin resistance in iLIRKO mice [82].

On the other hand, it has been described that IRA, but not IRB, favours basal glucose uptake through its specific association with endogenous GLUT1/2 in murine hepatocytes and beta cells [92, 93]. These associations produce an integrated system of insulin-dependent interactions that is highly sensitive to glucose concentration [94]. Moreover, IRA is more efficient than IRB at promoting glycogen synthesis and storage in murine hepatocytes [95] (Figure 3).

Obesity is the most common condition associated with insulin resistance. Therefore, the improvement of insulin resistance induced by weight loss obtained by a very low-calorie diet or bariatric surgery results in an increase in IRB in adipose tissue [96]. Changes in the IRA/IRB ratio in favour of the more metabolically active IRB are often related to a reduction in circulating insulin levels and should contribute to improve insulin sensitivity [77] (Figure 3).

### 7.2. Vascular Complications

Type 2 diabetes, metabolic syndrome, and obesity are well-known risk factors for atherosclerosis, in part because of insulin resistance and/or the hyperinsulinemia that are frequently present in these pathologies [97, 98]. On the other hand, accelerated atherosclerosis in diabetic patients has been associated with the fact that vascular smooth muscle cells from these patients present a significantly high rate of proliferation, adhesion, and migration [99]. Several authors have studied the role of IGF-IR in the different stages of the atherosclerotic process. However, the profile of IR isoforms has been less studied. In this sense, it has been described that in early atherosclerosis, IGF-IR may contribute to atherosclerotic progression by mediating the proatherogenic actions of IGFs. Thus, mRNA IGF-IR expression was enhanced in VSMCs of atherosclerotic plaques of asymptomatic patients compared to those from symptomatic patients [100]. Increased IGF-IR expression was also reported in VSMCs-derived foam cells present in rabbit atherosclerotic lesions [101] and in the aorta of 24-week-old ApoE^−/−^ mice [100]. In the same line, a recent study showed that monocyte/macrophage-specific IGF-IR deficiency in ApoE^−/−^ mice significantly increased the formation of atherosclerotic lesion and modified the composition of the monocyte/macrophage to a less stable phenotype [102]. Since IGF-I is a potent mitogen for VSMCs, numerous studies in experimental atherosclerosis models suggest that IGF-I promotes vascular hyperplasia by promoting neointimal growth [103]. In particular, the overexpression of IGF-I in the VSMCs of ApoE^−/−^ mice increased the number of VSMCs in the atherosclerotic plaque, the collagen content, and the fibrous capsule area, favouring plaque stability [104]. Regarding IGF-II, it has been proposed as an essential promoter of the growth of atherosclerotic lesions in the ApoE^−/−^ model and local overexpression is capable to induce the appearance of focal thickening of the intima [105]. In this sense, a significant increase in the expression of IRA in the aorta of 24-week-old ApoE^−/−^ mice has been described, which may favour the proatherogenic actions of IGF-II. In addition, gene expression of IRA, IGF-IR, and IGFs has been reported to be markedly increased in the middle layer, mainly composed of VSMCs, of human aortas with early atherosclerotic lesions [106] (Figure 3).

It has been established that the IR isoforms and the IGF-IR have a differential role in early and advanced atherosclerosis, since they favour atherogenesis in the initial stages while preventing instability and rupture of atherosclerotic plaques [106]. In more advanced phases, where local IGF-II action might prevent plaque weakening by promoting the proliferation of VSMCs in the intima [105], there is a decrease in the IRA/IRB ratio and less IGF-II actions, and as a consequence, it would be favouring the apoptosis and instability of atherosclerotic lesions.

Defects in insulin signalling at the level of the IR have been implicated in other complications such as diabetic nephropathy [107] and retinopathy [108]. However, the role of IR isoforms in these pathologies has not been described yet.

## 8. Role of IR Isoforms in the New Therapeutic Approaches for Type 2 Diabetes Treatment

Future research with new IR modulators might be considered as possible targets to improve the treatment of diabetes and its associated complications. For instance, insulin analogs are orthosteric variants of native insulin developed to efficiently mimic physiologic insulin secretion and achieve improved glycemic control [109, 110]. However, due to the structural differences with native insulin, insulin analogs may interact with IR isoforms and the IGF-IR with different binding affinities and dissociation rates, which may affect the activation of downstream signalling cascades. In theory, they may trigger imbalanced mitogenic effects compared to native insulin [109, 111]. Therefore, since insulin analogs are used in large diabetic populations, all new insulin analogs have to be tested for their mitogenic potential risk *in vitro* and *in vivo*. In addition to insulin analogs, there are many other IR modulators, and one possible approach is to design IR modulators that would induce conformational changes of the receptor different from the one induced by the natural ligand to stimulate selective responses in terms of time, intensity, and quality of downstream signals. For this reason, a number of new IR ligands able to separate the metabolic from the mitogenic IR actions have been studied in the last decade (i.e., orthosteric IR antibodies, antagonist antibodies, small synthetic peptides, and aptamers). The conformational changes induced by both orthosteric and allosteric ligands can differentially activate the postreceptor signalling pathways and insulin-dependent gene expression. This may result in important advantages with great relevance in clinical terms. However, despite these advances in generating IR modulators, the fine-tuning of IR signalling is far from being reached. Many issues require further investigation, such as the structure/function interactions, optimization of the complex network of intracellular signalling, tissue specificity of selective IR activation/inhibition, and the long-term consequences in the whole organism [112, 113].

## 9. Conclusions

This review discussed specifically the role of IR isoforms as well as IGF-IR in diabetes and its associated complications as obesity and atherosclerosis. It has been described an increase of IRA/IRB ratio conferring a proliferative and migratory advantage to *β*-cells or vascular smooth muscle cells of media layer of aorta and favouring IGF-II actions with a decrease of insulin signalling in white adipocytes of obese patients. These mechanisms contribute to hyperglycemia, hyperinsulinemia, obesity, and vascular complications associated to diabetes. Finally, future research with new IR modulators might be considered as possible targets to improve the treatment of diabetes and its associated complications.
